# Supplementary material for: Maternal microchimerism at birth associates with reduced odds of non-malarial fever and respiratory tract infections in Tanzanian children
Source: PLOS Glob Public Health. 2026 May 13;6(5):e0006439. doi: 10.1371/journal.pgph.0006439 (PMC13170846; doi:10.1371/journal.pgph.0006439)
Supplement: S1 Appendix — Table A the estimated associations between maternal microchimerism (MMc) and symptomatic infections in strata of age. Odds ratios (ORs) with 95% confidence intervals (CIs) are from Generalized Estimating Equation models (n = 37 children with no detected MMc*, 14 children with any detected MMc). Table B The estimated joint effect of maternal microchimerism (MMc) and age period on probability of symptomatic infections. Odds ratios (ORs) with 95% confidence intervals (CIs) are from Generalized Estimating Equation models (n = 37 children with no detected MMc*, 14 children with any detected MMc). Table C Odds ratios (ORs) with 95% confidence intervals (CIs) for symptomatic infections according to detected maternal microchimerism (MMc) in cord blood. The results are from Generalized Estimating Equation models (n = 37 children with no detected MMc*, 14 children with any detected MMc). The analyses are restricted to routine study visits. Table D Odds ratios (ORs) with 95% confidence intervals (CIs) for symptomatic infections according to detected maternal microchimerism (MMc) in cord blood. The results are from Generalized Estimating Equation models (n = 37 children with no detected MMc*, 14 children with any detected MMc). Recurrent infections within 7 days were excluded. Table E Odds ratios (ORs) with 95% confidence intervals (CIs) for symptomatic infections according to detected maternal microchimerism (MMc) in cord blood. The results are from Generalized Estimating Equation models (n = 37 children with no detected MMc*, 14 children with any detected MMc). Infections within 7 days of a malaria diagnosis were excluded if the child was positive for parasitemia. (DOCX) [file pgph.0006439.s001.docx]

**Assessment of effect measure modification by age**

***Table A*** *The estimated associations between maternal microchimerism (MMc) and symptomatic infections in strata of age. Odds ratios (ORs) with 95% confidence intervals (CIs) are from Generalized Estimating Equation models (n=37 children with no detected MMc*, 14 children with any detected MMc).*

| MMc test result | Unadjusted | | Adjusted for number of cells tested and placental malaria | | Additionally adjusted for maternal age at delivery, birth weight*, and months of exclusive breastfeeding | |
| --- | --- | --- | --- | --- | --- | --- |
|  | n visits  (n events) | OR (95% CI) | n visits  (n events) | OR (95% CI) | n visits  (n events) | OR (95% CI) |
| **Any respiratory tract infection, diarrhea, or fever** | | | | | | |
| Age 0 to 1 year | | | | | | |
| Not detected | 826 (162) | 1 (reference) | 826 (162) | 1 (reference) | 802 (157) | 1 (reference) |
| Detected | 335 (53) | 0.77 (0.55-1.08) | 335 (53) | 0.74 (0.52-1.04) | 315 (52) | 0.73 (0.50-1.07) |
| Age 1+ to 2 years | | | | | | |
| Not detected | 422 (78) | 1 (reference) | 422 (78) | 1 (reference) | 410 (74) | 1 (reference) |
| Detected | 150 (26) | 0.92 (0.57-1.51) | 150 (26) | 0.95 (0.58-1.54) | 150 (26) | 0.96 (0.58-1.58) |
| Age 2+ years | | | | | | |
| Not detected | 509 (60) | 1 (reference) | 509 (60) | 1 (reference) | 496 (60) | 1 (reference) |
| Detected | 143 (13) | 0.75 (0.40-1.41) | 143 (13) | 0.76 (0.40-1.43) | 143 (13) | 0.73 (0.37-1.46) |
| **Lower respiratory tract infection** | | | | | | |
| Age 0 to 1 year | | | | | | |
| Not detected | 826 (50) | 1 (reference) | 826 (50) | 1 (reference) | 802 (48) | 1 (reference) |
| Detected | 335 (16) | 0.78 (0.44-1.39) | 335 (16) | 0.77 (0.43-1.36) | 315 (16) | 0.90 (0.51-1.58) |
| Age 1+ to 2 years | | | | | | |
| Not detected | 422 (14) | 1 (reference) | 422 (14) | 1 (reference) | 410 (14) | 1 (reference) |
| Detected | 150 (3) | 0.59 (0.17-2.10) | 150 (3) | 0.60 (0.18-2.04) | 150 (3) | *NA* |
| Age 2+ years | | | | | | |
| Not detected | 509 (12) | 1 (reference) | 509 (12) | 1 (reference) | 496 (12) | 1 (reference) |
| Detected | 143 (1) | 0.29 (0.04-2.26) | 143 (1) | 0.27 (0.04-2.09) | 143 (1) | *NA* |
| **Upper respiratory tract infection** | | | | | | |
| Age 0 to 1 year | | | | | | |
| Not detected | 826 (96) | 1 (reference) | 826 (96) | 1 (reference) | 802 (93) | 1 (reference) |
| Detected | 335 (29) | 0.72 (0.47-1.12) | 335 (29) | 0.67 (0.43-1.04) | 315 (29) | 0.61 (0.38-1.00) |
| Age 1+ to 2 years | | | | | | |
| Not detected | 422 (51) | 1 (reference) | 422 (51) | 1 (reference) | 410 (47) | 1 (reference) |
| Detected | 150 (14) | 0.75 (0.40-1.40) | 150 (14) | 0.74 (0.40-1.38) | 150 (14) | 0.69 (0.35-1.34) |
| Age 2+ years | | | | | | |
| Not detected | 509 (34) | 1 (reference) | 509 (34) | 1 (reference) | 496 (34) | 1 (reference) |
| Detected | 143 (10) | 1.05 (0.51-2.18) | 143 (10) | 1.07 (0.52-2.21) | 143 (10) | *NA* |
| **Diarrhea** | | | | | | |
| Age 0 to 1 year | | | | | | |
| Not detected | 826 (14) | 1 (reference) | 826 (14) | 1 (reference) | 802 (14) | 1 (reference) |
| Detected | 335 (10) | 1.78 (0.79-4.05) | 335 (10) | 1.85 (0.79-4.29) | 315 (9) | 1.76 (0.66-4.70) |
| Age 1+ to 2 years | | | | | | |
| Not detected | 422 (7) | 1 (reference) | 422 (7) | 1 (reference) | 410 (7) | 1 (reference) |
| Detected | 150 (6) | 2.47 (0.82-7.44) | 150 (6) | 2.80 (1.03-7.60) | 150 (6) | 3.09 (1.18-8.08) |
| Age 2+ years | | | | | | |
| Not detected | 509 (10) | 1 (reference) | 509 (10) | 1 (reference) | 496 (10) | 1 (reference) |
| Detected | 143 (2) | 0.71 (0.15-3.27) | 143 (2) | 0.73 (0.16-3.33) | 143 (2) | 0.37 (0.05-2.92) |

*One child tested MMc negative had missing information on birth weight and was omitted from the fully adjusted analysis.

***Table B*** *The estimated joint effect of maternal microchimerism (MMc) and age period on probability of symptomatic infections. Odds ratios (ORs) with 95% confidence intervals (CIs) are from Generalized Estimating Equation models (n=37 children with no detected MMc*, 14 children with any detected MMc).*

| MMc test result | Unadjusted | | Adjusted for number of cells tested and placental malaria | | Additionally adjusted for maternal age at delivery, birth weight*, and months of exclusive breastfeeding | |
| --- | --- | --- | --- | --- | --- | --- |
|  | n visits  (n events) | OR (95% CI) | n visits  (n events) | OR (95% CI) | n visits  (n events) | OR (95% CI) |
| **Any respiratory tract infection, diarrhea, or fever** | | | | | | |
| Age 0 to 1 year | | | | | | |
| Not detected | 826 (162) | 1 (reference) | 826 (162) | 1 (reference) | 802 (157) | 1 (reference) |
| Detected | 335 (53) | 0.77 (0.55-1.08) | 335 (53) | 0.76 (0.54-1.06) | 315 (52) | 0.76 (0.53-1.09) |
| Age 1+ to 2 years | | | | | | |
| Not detected | 422 (78) | 0.93 (0.69-1.25) | 422 (78) | 0.93 (0.69-1.25) | 410 (74) | 0.91 (0.67-1.24) |
| Detected | 150 (26) | 0.86 (0.54-1.36) | 150 (26) | 0.84 (0.53-1.33) | 150 (26) | 0.81 (0.51-1.28) |
| Age 2+ years | | | | | | |
| Not detected | 509 (60) | 0.55 (0.40-0.75) | 509 (60) | 0.55 (0.40-0.76) | 496 (60) | 0.58 (0.42-0.80) |
| Detected | 143 (13) | 0.41 (0.23-0.74) | 143 (13) | 0.41 (0.22-0.74) | 143 (13) | 0.40 (0.22-0.74) |
| **Respiratory tract infection (upper or lower)** | | | | | | |
| Age 0 to 1 year | | | | | | |
| Not detected | 826 (146) | 1 (reference) | 826 (146) | 1 (reference) | 802 (141) | 1 (reference) |
| Detected | 335 (45) | 0.72 (0.50-1.04) | 335 (45) | 0.70 (0.49-1.00) | 315 (45) | 0.71 (0.49-1.04) |
| Age 1+ to 2 years | | | | | | |
| Not detected | 422 (65) | 0.85 (0.62-1.17) | 422 (65) | 0.84 (0.61-1.16) | 410 (61) | 0.82 (0.59-1.14) |
| Detected | 150 (17) | 0.60 (0.35-1.02) | 150 (17) | 0.57 (0.33-0.97) | 150 (17) | 0.54 (0.32-0.93) |
| Age 2+ years | | | | | | |
| Not detected | 509 (46) | 0.46 (0.33-0.66) | 509 (46) | 0.46 (0.33-0.66) | 496 (46) | 0.49 (0.34-0.70) |
| Detected | 143 (11) | 0.39 (0.20-0.74) | 143 (11) | 0.57 (0.33-0.97) | 143 (11) | 0.38 (0.20-0.73) |
| **Lower respiratory tract infection** | | | | | | |
| Age 0 to 1 year | | | | | | |
| Not detected | 826 (50) | 1 (reference) | 826 (50) | 1 (reference) | 802 (48) | 1 (reference) |
| Detected | 335 (16) | 0.78 (0.44-1.39) | 335 (16) | 0.76 (0.43-1.35) | 315 (16) | 0.92 (0.53-1.62) |
| Age 1+ to 2 years | | | | | | |
| Not detected | 422 (14) | 0.53 (0.29-0.97) | 422 (14) | 0.53 (0.29-0.97) | 410 (14) | 0.55 (0.30-1.01) |
| Detected | 150 (3) | 0.32 (0.10-1.03) | 150 (3) | 0.31 (0.10-1.00) | 150 (3) | 0.34 (0.11-1.11) |
| Age 2+ years | | | | | | |
| Not detected | 509 (12) | 0.37 (0.20-0.71) | 509 (12) | 0.38 (0.20-0.71) | 496 (12) | 0.37 (0.19-0.71) |
| Detected | 143 (1) | 0.11 (0.01-0.80) | 143 (1) | 0.11 (0.01-0.78) | 143 (1) | 0.12 (0.02-0.89) |
| **Upper respiratory tract infection** | | | | | | |
| Age 0 to 1 year | | | | | | |
| Not detected | 826 (96) | 1 (reference) | 826 (96) | 1 (reference) | 802 (93) | 1 (reference) |
| Detected | 335 (29) | 0.72 (0.47-1.11) | 335 (29) | 0.69 (0.45-1.07) | 315 (29) | 0.66 (0.42-1.05) |
| Age 1+ to 2 years | | | | | | |
| Not detected | 422 (51) | 1.05 (0.73-1.50) | 422 (51) | 1.04 (0.72-1.50) | 410 (47) | 1.00 (0.69-1.46) |
| Detected | 150 (14) | 0.78 (0.43-1.41) | 150 (14) | 0.75 (0.41-1.35) | 150 (14) | 0.67 (0.37-1.22) |
| Age 2+ years | | | | | | |
| Not detected | 509 (34) | 0.54 (0.36-0.82) | 509 (34) | 0.55 (0.36-0.82) | 496 (34) | 0.59 (0.39-0.90) |
| Detected | 143 (10) | 0.57 (0.29-1.13) | 143 (10) | 0.56 (0.28-1.10) | 143 (10) | 0.54 (0.27-1.09) |
| **Diarrhea** | | | | | | |
| Age 0 to 1 year | | | | | | |
| Not detected | 826 (14) | 1 (reference) | 826 (14) | 1 (reference) | 802 (14) | 1 (reference) |
| Detected | 335 (10) | 1.78 (0.78-4.06) | 335 (10) | 1.88 (0.81-4.32) | 315 (9) | 1.75 (0.70-4.40) |
| Age 1+ to 2 years | | | | | | |
| Not detected | 422 (7) | 0.98 (0.39-2.44) | 422 (7) | 0.97 (0.39-2.43) | 410 (7) | 0.98 (0.40-2.45) |
| Detected | 150 (6) | 2.42 (0.91-6.39) | 150 (6) | 2.57 (0.97-6.79) | 150 (6) | 2.50 (0.92-6.78) |
| Age 2+ years | | | | | | |
| Not detected | 509 (10) | 1.16 (0.51-2.64) | 509 (10) | 1.17 (0.52-2.66) | 496 (10) | 1.19 (0.52-2.70) |
| Detected | 143 (2) | 0.82 (0.18-3.66) | 143 (2) | 0.87 (0.20-3.85) | 143 (2) | 0.83 (0.19-3.61) |
| **Fever** | | | | | | |
| Age 0 to 1 year | | | | | | |
| Not detected | 826 (22) | 1 (reference) | 826 (22) | 1 (reference) | 802 (22) | 1 (reference) |
| Detected | 335 (2) | 0.22 (0.05-0.94) | 335 (2) | 0.23 (0.05-1.00) | 315 (2) | 0.25 (0.06-1.10) |
| Age 1+ to 2 years | | | | | | |
| Not detected | 422 (12) | 1.07 (0.52-2.18) | 422 (12) | 1.08 (0.53-2.20) | 410 (12) | 1.07 (0.52-2.17) |
| Detected | 150 (5) | 1.26 (0.47-3.38) | 150 (5) | 1.34 (0.50-3.61) | 150 (5) | 1.31 (0.47-3.64) |
| Age 2+ years | | | | | | |
| Not detected | 509 (16) | 1.19 (0.62-2.28) | 509 (16) | 1.18 (0.61-2.26) | 496 (16) | 1.14 (0.59-2.23) |
| Detected | 143 (0) | *NA* | 143 (0) | *NA* | 143 (0) | *NA* |

*One child tested MMc negative had missing information on birth weight and was omitted from the fully adjusted analysis.

**Restriction to routine visits**

***Table C*** *Odds ratios (ORs) with 95% confidence intervals (CIs) for symptomatic infections according to detected maternal microchimerism (MMc) in cord blood. The results are from Generalized Estimating Equation models (n=37 children with no detected MMc*, 14 children with any detected MMc). The analyses are restricted to routine study visits.*

| MMc test result | Unadjusted | | Adjusted for number of cells tested and placental malaria | | Additionally adjusted for maternal age at delivery, birth weight*, and months of exclusive breastfeeding | |
| --- | --- | --- | --- | --- | --- | --- |
|  | n visits  (n events) | OR (95% CI) | n visits  (n events) | OR (95% CI) | n visits  (n events) | OR (95% CI) |
| **Any respiratory tract infection, diarrhea, or fever** | | | | | | |
| Not detected | 1417 (203) | 1 (reference) | 1417 (203) | 1 (reference) | 1376 (195) | 1 (reference) |
| Detected | 537 (65) | 0.82 (0.61-1.11) | 537 (65) | 0.79 (0.59-1.07) | 517 (64) | 0.75 (0.54-1.03) |
| **Respiratory tract infections (upper or lower)** | | | | | | |
| Not detected | 1417 (191) | 1 (reference) | 1417 (191) | 1 (reference) | 1376 (183) | 1 (reference) |
| Detected | 537 (60) | 0.81 (0.59-1.10) | 537 (60) | 0.77 (0.57-1.05) | 517 (60) | 0.74 (0.53-1.04) |
| **Lower respiratory tract infections** | | | | | | |
| Not detected | 1417 (38) | 1 (reference) | 1417 (38) | 1 (reference) | 1376 (36) | 1 (reference) |
| Detected | 537 (12) | 0.83 (0.43-1.60) | 537 (12) | 0.82 (0.43-1.58) | 517 (12) | 0.94 (0.49-1.80) |
| **Upper respiratory tract infections** | | | | | | |
| Not detected | 1417 (153) | 1 (reference) | 1417 (153) | 1 (reference) | 1376 (147) | 1 (reference) |
| Detected | 537 (48) | 0.81 (0.58-1.14) | 537 (48) | 0.77 (0.55-1.08) | 517 (48) | 0.71 (0.49-1.03) |
| **Diarrhea** | | | | | | |
| Not detected | 1417 (11) | 1 (reference) | 1417 (11) | 1 (reference) | 1376 (11) | 1 (reference) |
| Detected | 537 (7) | 1.69 (0.65-4.38) | 537 (7) | 1.74 (0.66-4.61) | 517 (6) | 1.36 (0.44-4.18) |
| **Fever** | | | | | | |
| Not detected | 1417 (14) | 1 (reference) | 1417 (14) | 1 (reference) | 1376 (14) | 1 (reference) |
| Detected | 537 (2) | 0.37 (0.08-1.65) | 537 (2) | 0.40 (0.09-1.80) | 517 (2) | *NA* |

*One child tested MMc negative had missing information on birth weight and was omitted from the fully adjusted analysis.

**Quarantine of 7 days for recurrent infections**

***Table D*** *Odds ratios (ORs) with 95% confidence intervals (CIs) for symptomatic infections according to detected maternal microchimerism (MMc) in cord blood. The results are from Generalized Estimating Equation models (n=37 children with no detected MMc*, 14 children with any detected MMc). Recurrent infections within 7 days were excluded.*

| MMc test result | Unadjusted | | Adjusted for number of cells tested and placental malaria | | Additionally adjusted for maternal age at delivery, birth weight*, and months of exclusive breastfeeding | |
| --- | --- | --- | --- | --- | --- | --- |
|  | n visits  (n events) | OR (95% CI) | n visits  (n events) | OR (95% CI) | n visits  (n events) | OR (95% CI) |
| **Any respiratory tract infection, diarrhea, or fever** | | | | | | |
| Not detected | 1757 (274) | 1 (reference) | 1757 (274) | 1 (reference) | 1708 (266) | 1 (reference) |
| Detected | 628 (87) | 0.87 (0.67-1.13) | 628 (87) | 0.85 (0.66-1.11) | 608 (86) | 0.84 (0.63-1.11) |
| **Respiratory tract infections (upper or lower)** | | | | | | |
| Not detected | 1757 (240) | 1 (reference) | 1757 (240) | 1 (reference) | 1708 (232) | 1 (reference) |
| Detected | 628 (68) | 0.77 (0.58-1.02) | 628 (68) | 0.74 (0.56-0.98) | 608 (68) | 0.72 (0.53-0.98) |
| **Lower respiratory tract infections** | | | | | | |
| Not detected | 1757 (72) | 1 (reference) | 1757 (72) | 1 (reference) | 1708 (70) | 1 (reference) |
| Detected | 628 (19) | 0.73 (0.44-1.22) | 628 (19) | 0.72 (0.43-1.20) | 608 (19) | 0.83 (0.50-1.37) |
| **Upper respiratory tract infections** | | | | | | |
| Not detected | 1757 (179) | 1 (reference) | 1757 (179) | 1 (reference) | 1708 (172) | 1 (reference) |
| Detected | 628 (53) | 0.81 (0.59-1.12) | 628 (53) | 0.78 (0.57-1.07) | 608 (53) | 0.72 (0.50-1.02) |
| **Diarrhea** | | | | | | |
| Not detected | 1757 (31) | 1 (reference) | 1757 (31) | 1 (reference) | 1708 (31) | 1 (reference) |
| Detected | 628 (18) | 1.64 (0.91-2.96) | 628 (18) | 1.73 (0.96-3.10) | 608 (17) | 1.63 (0.85-3.13) |
| **Fever** | | | | | | |
| Not detected | 1757 (45) | 1 (reference) | 1757 (45) | 1 (reference) | 1708 (45) | 1 (reference) |
| Detected | 628 (7) | 0.43 (0.19-0.96) | 628 (7) | 0.45 (0.20-1.01) | 608 (7) | 0.49 (0.21-1.14) |

*One child tested MMc negative had missing information on birth weight and was omitted from the fully adjusted analysis.

**Quarantine of 7 days following every malaria diagnosis if positive for parasitemia**

***Table E*** *Odds ratios (ORs) with 95% confidence intervals (CIs) for symptomatic infections according to detected maternal microchimerism (MMc) in cord blood. The results are from Generalized Estimating Equation models (n=37 children with no detected MMc*, 14 children with any detected MMc). Infections within 7 days of a malaria diagnosis were excluded if the child was positive for parasitemia.*

| MMc test result | Unadjusted | | Adjusted for number of cells tested and placental malaria | | Additionally adjusted for maternal age at delivery, birth weight*, and months of exclusive breastfeeding | |
| --- | --- | --- | --- | --- | --- | --- |
|  | n visits  (n events) | OR (95% CI) | n visits  (n events) | OR (95% CI) | n visits  (n events) | OR (95% CI) |
| **Any respiratory tract infection, diarrhea, or fever** | | | | | | |
| Not detected | 1809 (328) | 1 (reference) | 1809 (328) | 1 (reference) | 1759 (318) | 1 (reference) |
| Detected | 663 (116) | 0.96 (0.76-1.21) | 663 (116) | 0.94 (0.75-1.19) | 643 (115) | 0.92 (0.72-1.19) |
| **Respiratory tract infections (upper or lower)** | | | | | | |
| Not detected | 1809 (284) | 1 (reference) | 1809 (284) | 1 (reference) | 1759 (274) | 1 (reference) |
| Detected | 663 (96) | 0.91 (0.71-1.17) | 663 (96) | 0.88 (0.69-1.13) | 643 (96) | 0.86 (0.66-1.13) |
| **Lower respiratory tract infections** | | | | | | |
| Not detected | 1809 (99) | 1 (reference) | 1809 (99) | 1 (reference) | 1759 (96) | 1 (reference) |
| Detected | 663 (42) | 1.17 (0.81-1.70) | 663 (42) | 1.14 (0.78-1.65) | 643 (42) | 1.25 (0.85-1.85) |
| **Upper respiratory tract infections** | | | | | | |
| Not detected | 1809 (185) | 1 (reference) | 1809 (185) | 1 (reference) | 1759 (178) | 1 (reference) |
| Detected | 663 (54) | 0.78 (0.57-1.07) | 663 (54) | 0.75 (0.55-1.03) | 643 (54) | 0.70 (0.49-0.99) |
| **Diarrhea** | | | | | | |
| Not detected | 1809 (32) | 1 (reference) | 1809 (32) | 1 (reference) | 1759 (32) | 1 (reference) |
| Detected | 663 (18) | 1.55 (0.86-2.78) | 663 (18) | 1.63 (0.91-2.91) | 643 (17) | 1.51 (0.80-2.87) |
| **Fever** | | | | | | |
| Not detected | 1809 (53) | 1 (reference) | 1809 (53) | 1 (reference) | 1759 (53) | 1 (reference) |
| Detected | 663 (15) | 0.77 (0.43-1.37) | 663 (15) | 0.79 (0.44-1.40) | 643 (15) | 0.82 (0.45-1.52) |

*One child tested MMc negative had missing information on birth weight and was omitted from the fully adjusted analysis.
