# Supplementary material for: Maternal microchimerism at birth associates with reduced odds of non-malarial fever and respiratory tract infections in Tanzanian children
Source: PLOS Glob Public Health. 2026 May 13;6(5):e0006439. doi: 10.1371/journal.pgph.0006439 (PMC13170846; doi:10.1371/journal.pgph.0006439)
Supplement: Attachment — Submitted filename: PGPH-D-25-02597_reviewer_18122025.docx [file pgph.0006439.s003.docx]

Petersen et al. present a secondary analysis of a birth cohort comprising 52 children from Tanzania, describing an association between cord blood microchimerism and protection against respiratory tract infections and non-malaria febrile illness during the first four years of life. The authors are to be commended for leveraging a uniquely rich longitudinal dataset, with follow-up conducted every two weeks during the first two years of life and every four weeks thereafter, up to five years. The study is well conducted, the analyses are appropriate, and the manuscript is well written. However, some points require clarification or minor revision to improve transparency, interpretability, and completeness of the manuscript.

**Minor revisions**

**Methods**

- Please include the approval number.
- The manuscript states that the study was conducted between 2002 and 2006. Please specify the exact start and end dates (day and month), including recruitment and follow-up details.
- The methods used to assess maternal microchimerism should be described in greater detail in the current manuscript, including laboratory techniques, thresholds for positivity, quality control procedures, and any relevant validation references.
- Please expand the description of how symptomatic infection outcomes were defined. Specifically: (1) who made the diagnoses (study personnel vs. routine healthcare providers), (2) if there were ICD-10 or other standardized diagnostic codes used, and (3) if there were any diagnostic tests performed beyond malaria blood smears. In addition to including this information in the methods section, please comment on it in the discussion and address it in the limitations, as appropriate.
- Additionally, as all infection outcomes refer exclusively to symptomatic infections, please ensure this is stated explicitly and consistently throughout the manuscript, including the abstract.

**Results:**

- Please clearly report the number of mother–infant pairs included in the original Mother Offspring Malaria Study cohort and the number included in this secondary analysis. If not all participants were analysed, please explain the reasons for non-inclusion. I also suggest inclusion of a participant flow diagram to improve clarity.
- Table 1. Please report the prevalence of relevant maternal and child comorbidities (e.g., HIV infection, tuberculosis, malnutrition), as these may act as important confounders. If such data are unavailable, this limitation should be explicitly acknowledged and discussed.
- In line 182, the manuscript reports 330 respiratory tract infection events, including 96 lower RTIs and 234 upper RTIs, as well as 49 diarrhoea and 57 fever events. However, Figure 1 refers to 392 symptomatic infections in total. This discrepancy is likely due to overlapping events, but this should be clearly explained in the text to avoid confusion.

**Figures:**

- The current resolution and visual quality of both figures are insufficient. Please improve figure quality and consider using lighter or more contrasting colours in Figure 1 to enhance readability, particularly for text within the figure.
