# Supplementary material for: Maternal microchimerism at birth associates with reduced odds of non-malarial fever and respiratory tract infections in Tanzanian children
Source: PLOS Glob Public Health. 2026 May 13;6(5):e0006439. doi: 10.1371/journal.pgph.0006439 (PMC13170846; doi:10.1371/journal.pgph.0006439)
Supplement: Attachment — Submitted filename: Response to Reviewers - 4Feb26.docx [file pgph.0006439.s005.docx]

Dear Dr. Howard,

Thank you for the opportunity to revise and resubmit our manuscript. We are pleased to hear that the reviewer finds that our work has merit, and we thank the reviewer for the constructive comments. We have revised the manuscript accordingly, which we feel has strengthened the manuscript.

In our resubmission, we include two versions of the revised document: a version with all changes marked in track changes and a clean version including revisions.

Below, we respond point-by-point to the comments.

**Editorial comments:**
1. Please provide separate figure files in .tif or .eps format.

*Response: Both figures have now been updated, provided in higher resolution, and uploaded in the suggested format. To accommodate the comment of the reviewer, we additionally adjusted the color scheme in Figure 1 to improve contrast and readability, particularly for the text elements.*

**Reviewer #1:**

Petersen et al. present a secondary analysis of a birth cohort comprising 52 children from Tanzania, describing an association between cord blood microchimerism and protection against respiratory tract infections and non-malaria febrile illness during the first four years of life. The authors are to be commended for leveraging a uniquely rich longitudinal dataset, with follow-up conducted every two weeks during the first two years of life and every four weeks thereafter, up to five years. The study is well conducted, the analyses are appropriate, and the manuscript is well written. However, some points require clarification or minor revision to improve transparency, interpretability, and completeness of the manuscript.

Minor revisions

**Methods:**
1. Please include the approval number.

*Response: We appreciate this feedback. The original Tanzanian Institutional Review Board did not assign a protocol number during the review of the original MOMS cohort, so it is not possible to add. We have added the US study number, and the text now reads: “The establishment of the cohort was approved by both United States (Western Institutional Review Board Study 1059357) and Tanzanian (National Institute for Medical Research, Medical Research Coordinating Committee) ethical review boards.” (p. 6, l. 108-110)*

2. The manuscript states that the study was conducted between 2002 and 2006. Please specify the exact start and end dates (day and month), including recruitment and follow-up details.

*Response: We have edited the manuscript to add the full dates. The text is updated to: “We present a secondary analysis of the Mother Offspring Malaria Study birth cohort conducted in Muheza, Tanzania between September 9^th^, 2002 and May 15^th^, 2006 (15).” (p. 6, l. 95-96)*

3. The methods used to assess maternal microchimerism should be described in greater detail in the current manuscript, including laboratory techniques, thresholds for positivity, quality control procedures, and any relevant validation references.

*Response: Thank you for this comment. We acknowledge that additional details are useful for the understanding and interpretation of the findings. We expanded the details of microchimerism assessment which now reads: “Level of cord blood MMc was previously measured (14). Briefly, MMc was identified by targeting a non-shared, non-inherited maternal polymorphism in genomic DNA (gDNA) extracted from cord blood. When informative human leucocyte antigen (HLA) polymorphisms were not available, four non‑HLA loci (ATIII, TG, GSTT1, TNN) targeting insertion/deletion/substitution variants were used to identify a maternal-specific marker. Maternal‑specific polymorphisms were then amplified from cord‑blood gDNA using qPCR assays with a sensitivity of 1 maternal genomic equivalent (gEq) in 20 000 background infant gEq. Each assay included a polymorphism‑specific calibration curve to quantify MMc. Samples were also tested for the nonpolymorphic β‑globin gene (HBB), with an HBB calibration curve used to determine total gEq per reaction. Only samples with ≥10⁴ expected gEq were included. MMc was modeled both as a binary predictor (no vs. any MMc) and as a normalized continuous predictor (MMc gEq per 10⁵ gEq tested) (14).” (p. 7, l. 113-122)*

4. Please expand the description of how symptomatic infection outcomes were defined. Specifically: (1) who made the diagnoses (study personnel vs. routine healthcare providers), (2) if there were ICD-10 or other standardized diagnostic codes used, and (3) if there were any diagnostic tests performed beyond malaria blood smears. In addition to including this information in the methods section, please comment on it in the discussion and address it in the limitations, as appropriate.

*Response: We appreciate this thoughtful comment. To clarify, no system of standardized diagnostic codes was used and limited diagnostic tests were performed beyond malaria blood smears. We have expanded the description in the Study Population section to provide a clearer and more detailed explanation of how symptomatic infections were defined**: “Upon each study visit, symptoms of infectious disease were registered by the study personnel (clinician or village health worker) on exam or by parental report. All LRTI diagnoses were validated on physical exam by a study clinician, whereas URTI and diarrhea were defined by the presence of consistent symptoms reported by the study personnel or the parents. Fever was objectively measured by the study personnel and defined as a temperature >38°C.” (p. 7, l. 124-128).*

*In addition, we have added the following paragraph on the limitations to the discussion: ”Second, the certainty of our outcome measures may vary across conditions. Fever was measured objectively by trained study personnel, and LRTI diagnoses were validated by a study clinician. In contrast, URTI and diarrhea diagnoses relied more heavily on parental reports, and no international diagnosis classification codes were used in the study. These differences may reduce the certainty of some outcome measures. Differential misclassification is unlikely, as neither parents nor study personnel were aware of infants’ MMc status, and we are not aware of any plausible indirect mechanism linking MMc to infection reporting or diagnosis. Any resulting bias would therefore be expected to attenuate associations toward the null. Replication in cohorts with more standardized infection measures would help confirm our findings.” (p. 17, l. 262 – p. 18, l. 269)*

5. Additionally, as all infection outcomes refer exclusively to symptomatic infections, please ensure this is stated explicitly and consistently throughout the manuscript, including the abstract.

*Response: Thank you for this feedback. We have edited the manuscript throughout to indicate that we were assessing odds of symptomatic infections as our outcome measure.*

**Results:**
6. Please clearly report the number of mother–infant pairs included in the original Mother Offspring Malaria Study cohort and the number included in this secondary analysis. If not all participants were analysed, please explain the reasons for non-inclusion. I also suggest inclusion of a participant flow diagram to improve clarity.

*Response: The original MOMS cohort included n=882 maternal-infant pairs. Microchimerism levels at birth were measured in only a subset of individuals as presented in our manuscript (Harrington, JID, 2017), based on availability of placental histology for PM categorization (n = 270), available paired maternal cord samples (n = 77), informative typing for a maternal-specific allele (n = 63), and sufficient CB sample size to assess MMc (n = 52). While this represents only a fraction of the original cohort, there should not have been bias in those who were sampled versus not based on these sample attrition characteristics. Unfortunately, because the original cohort was enrolled many years ago, there were no additional resources available to expand the number of individuals assessed for microchimerism. However, given that n=52 infants yielded 2,385 visits for consideration, we were still able to have the power to detect meaningful associations with clinical outcomes.*

*Because of the multi-step attrition of samples / individuals considered from the original cohort, which was dependent upon available samples at the time of microchimerism measurement, we feel that a participant flow diagram may actually add to confusion (as the loss of samples was purely technical and not based on anything to do with participants). As such, we have opted to not add a flow table but rather better clarify this information as added to the methods: p. 6, l. 95-101.*

7. Table 1. Please report the prevalence of relevant maternal and child comorbidities (e.g., HIV infection, tuberculosis, malnutrition), as these may act as important confounders. If such data are unavailable, this limitation should be explicitly acknowledged and discussed.

*Response: We appreciate this comment. The MOMS cohort only included women with an uncomplicated singleton pregnancy and without known chronic diseases such as HIV or tuberculosis. Similarly, no documented HIV, tuberculosis, or clinical malnutrition were noted in the included children. However, information on more subtle potential comorbidities was not collected during the course of the study. To clarify this point, we have added to the description of the study population to note that mothers were: “…free from chronic illness”, (p. 6, l. 98). In addition, we have added this as a potential limitation of the study in the discussion: “Fifth, predictors of cord blood MMc are not yet well understood, which complicates the identification of confounders such as additional unmeasured comorbidities. Although our alternatively adjusted models yielded largely similar results, unmeasured confounding is possible. Future studies should therefore investigate maternal and infant determinants of cord blood MMc to improve confounder identification and strengthen causal inference.” (p. 18, l. 279-283).*

*We would also note that infant comorbidities would be considered downstream of the exposure as MMc was measured at birth, where adjustment for intermediate variables will typically result in bias towards the null when aiming to estimate the total effect (PMID: 19525685).*

8. In line 182, the manuscript reports 330 respiratory tract infection events, including 96 lower RTIs and 234 upper RTIs, as well as 49 diarrhoea and 57 fever events. However, Figure 1 refers to 392 symptomatic infections in total. This discrepancy is likely due to overlapping events, but this should be clearly explained in the text to avoid confusion.

*Response: We apologize for the confusion. We rephrased the paragraph and hope the revised version is clearer: “The 52 children contributed 2,385 aparasitemic study visits, of which 1,993 were asymptomatic. Symptomatic infections were registered at 392 of these visits: 348 visits with a single symptomatic infection and 44 visits with two concurrent symptomatic infections, yielding a total of 436 infection events. These events comprised 330 RTI events (96 LRTI and 234 URTI), 49 diarrhea events, and 57 fever events (Fig 1).” (p. 12, l. 184-187).*

*We have also revised the figure text accordingly: “****Fig 1. Venn diagram of symptomatic non-malarial infections among aparasitemic visits.*** *This Venn diagram illustrates the distribution of aparasitemic study visits (n = 2,385) with and without symptomatic non-malarial infections. Symptomatic infections were registered at 392 visits: 348 visits with a single symptomatic infection and 44 with two concurrent symptomatic infections, yielding a total of 436 symptomatic infection events. Symptom categories include lower respiratory tract infection (LRTI, green), upper respiratory tract infection (URTI, blue), diarrhea (brown), and fever (red). Overlapping regions represent visits with multiple concurrent symptoms.” (p. 12, l. 188-193).*

**Figures:**
9. The current resolution and visual quality of both figures are insufficient. Please improve figure quality and consider using lighter or more contrasting colours in Figure 1 to enhance readability, particularly for text within the figure.

*Response: We appreciate this suggestion. Both figures have now been updated and provided in higher resolution, and the color scheme in Figure 1 has been adjusted to improve contrast and readability, particularly for the text elements.*

On behalf of the authors,

Whitney Harrington, MD PhD
